# Hughes-Stovin Syndrome and the Acute Management of Recurrent Pulmonary Aneurysms

**DOI:** 10.7759/cureus.28672

**Published:** 2022-09-01

**Authors:** Adam Cole, Anupa Mandava

**Affiliations:** 1 Internal Medicine, SUNY Upstate Medical University, Syracuse, USA

**Keywords:** autoimminue, hypercoagubility, behcet's disease, pulmonary aneurysm, hugh-stovin syndrome

## Abstract

Hughes-Stovin syndrome (HSS) is a rare autoimmune disease that is considered to be a variant of Behçet’s disease. It typically presents with multiple venous thrombi, thrombophlebitis, as well as pulmonary and bronchial aneurysms. The disease is typically associated with high morbidity and mortality in those diagnosed. This case discusses a 28-year-old male with HSS complicated by venous cardiac emboli and a mechanical valve on chronic anticoagulation. Despite medical and surgical management, the patient experienced continued progression of his pulmonary aneurysms and excessive bleeding, eventually requiring lung transplantation.

## Introduction

Hughes-Stovin syndrome is a rarely reported autoimmune disease that typically presents as multiple deep venous thrombi as well as pulmonary and bronchial aneurysms, mostly in young adult males [1]. The disease usually progresses over the course of several years with complications related to bleeding from pulmonary aneurysms [2]. Currently, the pathophysiology of the illness is not completely understood, however, the literature suggests that it could be a type of vasculitis that is considered to be an incomplete version of Behçet’s disease [1]. It is important to note, that while similar to Behçet’s disease, HSS lacks the aphthous and genital ulcers, making it a unique variant [1]. Another purported disease mechanism is hypercoagulability caused by autoimmune vasculitis [3]. Due to this hypercoagulable status, when the patient gets an infection, the preformed thrombi become a locus for bacteria. From there, the thrombi can become septic emboli, resulting in pulmonary aneurysms that are the hallmark of the disease [3]. This mechanism is supported by several case reports that indicate that infections preceded the formation of multiple pulmonic aneurysms [3]. Due to the rarity of the disease, there are currently no strict diagnostic criteria for HSS, and as such, it is typically only used as the primary diagnosis if the patient presents with all the previously mentioned symptoms and all other causes have been ruled out.

## Case presentation

A 28-year-old African American male was admitted to our facility due to an episode of hemoptysis and subsequent acute hypoxia. His documented clinical history began in 2014 when he immigrated to the United States from Kenya. In 2015, he developed a cough with sputum that was acid-fast bacillus (AFB) positive, resulting in him undergoing anti-TB treatment with RIPE therapy (rifapentine, isoniazid, pyrazinamide, and ethambutol) for six months. In 2018, the patient was found to have upper lobe cavitating pneumonia with mediastinal lymphadenopathy and began another six-month empiric treatment of RIPE therapy. However, at the time all cultures and mediastinal lymph node biopsy were AFB negative. Despite the negative cultures, treatment with RIPE therapy continued as upper lobe cavitating pneumonia with an elevated ESR was highly suspicious of recurrent TB clinically.

During his treatment, he was found to have a pulmonary artery aneurysm with arteriovenous clot, venous thromboembolism involving deep vein thrombosis, inferior vena cava thrombi, and intracardiac thrombi with nonbacterial thrombotic endocarditis of tricuspid valve. Due to the nonbacterial thrombotic endocarditis (NBTE), he underwent two separate valve replacements, including a mechanical tricuspid valve, and was started on chronic anticoagulation with warfarin. Surgical pathology and 16s rRNA analysis obtained during this period were all negative for an infectious source.

Given the negative infectious workup, the initial diagnosis of recurrent TB was called into question. An autoimmune workup was initiated, which found a Dilute Russell's viper venom time (dRVVT) test to be positive. Other serologies including lupus anticoagulant, B2 glycoprotein, and cardiolipin were performed to identify if the patient had antiphospholipid syndrome. However, all these additional serologies were found to be negative. Based on his clinical features, positive dRVVT, and other negative autoimmune serologies, the diagnosis of HSS was made. At the time, there was some suspicion that his symptoms could be caused by antiphospholipid syndrome or tuberculosis, as both have been associated with pulmonary artery aneurysms. However, given the extent of his symptoms, his negative infectious workup, and negative autoimmune serologies, the diagnosis of HSS was deemed to be more likely, particularly in the absence of classic Bechet's symptoms.

After establishing the diagnosis, the patient was started on cyclophosphamide and mycophenolate mofetil, as immunosuppression has shown some effectiveness in the literature [4]. Despite the immunosuppression, in 2020 the patient had a left lower lung lobectomy to control recurrent pulmonary aneurysms. Biopsies taken during the lobectomy showed capillaritis, hemorrhage, and multiple clots without any specific autoimmune features. In an attempt to cover both a potential antiphospholipid syndrome or HSS flare the patient received plasmapheresis, steroids, and infliximab. Continued monitoring for an infectious source was also performed given the patient’s history of TB. However, AFB cultures continued to be negative further solidifying the diagnosis of HSS.

Unfortunately, a few months later the patient developed recurrent bacterial pneumonia, likely secondary to his immunosuppression. The immunosuppression was halted, and the patient was admitted to the hospital for treatment. During his stay, he developed proteinuria, which was investigated with a renal biopsy. The culture of both his renal biopsy and bronchial aspirate grew mycobacterium chelonae, leading to a new diagnosis of disseminated mycobacterium chelonae. Treatment with amikacin, clarithromycin, and linezolid was initiated. During this period, his kidneys were also found to be co-infected with Candida albicans. Following treatment resolution, the patient developed drug-induced hepatitis, acute tubular necrosis, and pancreatitis, which were treated over another extended hospital stay.

**Figure 1:**
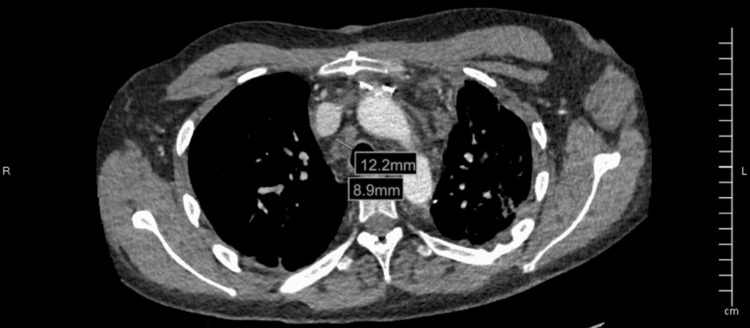
Right pulmonary artery aneurysm

**Figure 2:**
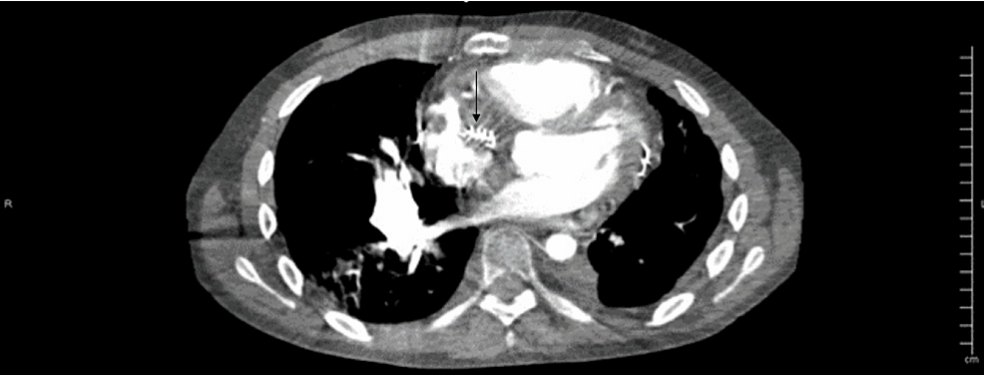
Right pulmonary artery intact coil embolization

**Figure 3:**
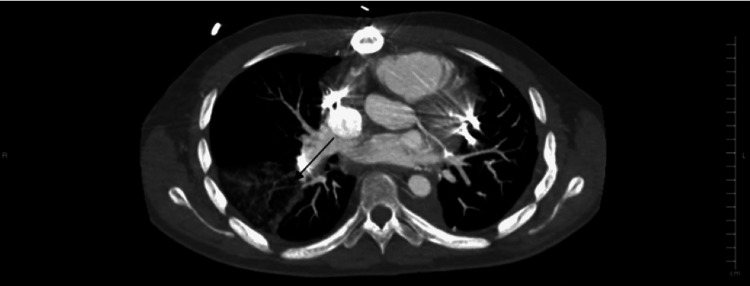
Wedge-shaped pulmonary infarct of the right lower lobe

One week prior to his most recent admission, the patient had been admitted to the medical ICU due to a separate episode of hemoptysis, where he underwent embolization and coiling for a right pulmonary artery aneurysm (Figure 1) and began a course of IV vancomycin and IV piperacillin/tazobactam for pneumonia. After completion of the treatment, the patient was discharged, however, he developed new-onset hemoptysis several days later leading to readmission. On the current admission, his troponin was elevated, and his INR was within the therapeutic range, however, due to the multiple episodes of hemoptysis, anticoagulation was held. A repeat CT angiography showed an intact coil embolization alongside a wedge-shaped pulmonary infarct of the right lower lobe (Figures 2-3). A previous coil was also found to be eroding into the airway and was confirmed via bronchoscopy. Coil packing was deemed too risky, as well as having a high likelihood of failure. Surgery was also ruled out as a treatment option, due to the previous history of left lower lung lobectomy and poor pulmonary reserve. Due to the progressive nature of his disease in spite of multiple management modalities and the high likelihood of new aneurysm formation causing bleeding and his need for chronic anticoagulation, a lung transplant was decided to be the only viable treatment option and he was transferred to a regional transplant center.

## Discussion

Hughes-Stovin syndrome (HSS) is a rare disease that is thought to be a variant of Bechet’s disease [1]. Due to the uncommon nature of Hughes-Stovin syndrome, there are no universal criteria for disease diagnosis [1]. Typically, HSS is a diagnosis of exclusion, but there are several key features that are usually associated with the illness. These include multiple deep venous thrombi, pulmonary/bronchial aneurysms, systemic phlebitis, and hemoptysis [1]. In the case of the previously presented patient, the diagnosis of HSS was only made after most other mechanisms for his presenting symptoms had been ruled out. Initially, it seemed that his symptoms may have been infectious in origin, as he had been previously diagnosed with tuberculosis. However, negative surgical pathology and 16 rRNA analysis indicated that it was unlikely that an infectious source was present. A similar extensive workup was performed to investigate autoimmune causes. Given the negative lupus anticoagulant, B2 glycoprotein, and cardiolipin, it was deemed unlikely that the cause was more routine autoimmune processes such as antiphospholipid syndrome. Despite this, the positive dRVVT indicated that some autoimmune process was likely involved. With this in mind, the diagnosis of HSS seemed to best explain all of the patient's presenting symptoms in the absence of another clear diagnosis.

In other patients with HSS, immune suppression has been shown to result in the stabilization of their pulmonary aneurysms [4]. However, in the case of this patient immune suppression did not result in long-term control. This failure to respond to therapy led to a left lower lobectomy to control his episodes of hemoptysis, as well as the implementation of a more robust immunosuppressive regimen. Unfortunately, this resulted in the patient developing sepsis, leading to the halt of immunosuppressive therapy. The other challenge with this case was adequately managing the patient’s need for anticoagulation due to his prosthetic heart valve and atrial thrombus in the setting of hemoptysis secondary to disease progression and recurrent pulmonary aneurysms. The continued use of anticoagulation could have also played a role in explaining why the patient had repeated episodes of hemoptysis despite initial immunosuppression. It also directly contributed to one of the primary sources of morbidity in this patient’s disease process, i.e. hemoptysis. This increased morbidity and the failure of both surgical and medical treatments led to the patient requiring a lung transplant. The eventual transplant points to the need for more research on management guidelines and weighing the usage of anticoagulation in the setting of mechanical heart valves, multiple venous thrombi, and bleeding complications from pulmonary aneurysms.

## Conclusions

Due to the chronic and unknown pathophysiology of Hughes-Stovin syndrome, treatment options are limited considering the high risk of bleeding from pulmonary aneurysms and simultaneous cardiac and venous thromboembolism. In our case, the need for anticoagulation with his history of mechanical tricuspid valves only made his treatment more difficult as his bleeding risk was extremely high. While there has been some success managing the disease using immunosuppression in conjunction with surgical resection and embolization, the end result of the disease course makes the need for lung transplant high in this patient population. Due to the complex course and complications of HSS, we need more studies to establish clear diagnostic criteria earlier in the disease course and to formulate appropriate management guidelines.
